# The Suppression of Depression as Multimediation: Psychiatric Diagnoses Under Myanmar's Military Dictatorship

**DOI:** 10.1007/s11013-025-09899-3

**Published:** 2025-03-23

**Authors:** Stefan Ecks

**Affiliations:** School of Social & Political Science, University of Edinburgh, 18 Buccleuch Place, Edinburgh, EH8 9LN UK

**Keywords:** Depression, Myanmar, Violence, Living value theory, Multimediation model of health

## Abstract

Myanmar has experienced decades of military dictatorship, civil wars, religious violence, economic crises, and natural disasters. While these conditions would suggest very high rates of depression and anxiety, government statistics report an exceptionally low depression rate of 0.00006%, compared to the global rate of 3.4%. This study combines analysis of epidemiological data, ethnographic observation of clinics, and in-depth interviews. I argue that Myanmar's low depression rates cannot be explained by the usual arguments about treatment gaps, lack of providers, or medication accessibility. Instead, I suggest that the military regime suppresses depression because it sees it as a form of political protest. While conditions like schizophrenia are readily diagnosed and treated as “purely biological,” mood disorders are suspect expressions of dissent. Through living value theory (LVT), I explore health as a process of multimediation. The dictatorship’s suppression of depression emerges as the strategic muting of medical interventions in favor of amplifying non-medical remediations.

## "I have never seen anyone with severe depression"

"I have never seen anyone with severe depression. It's not like that in Myanmar," Dr. Zaw said in an interview in 2020. Why are depression and other mood disorders said to be rare in a country that experienced decades of military dictatorship, interethnic violence, economic crises, and deadly natural disasters? By tracing psychotropic drugs and epidemiological data in Myanmar, I explore how the country's healthcare professionals explain the low incidence of depression and the low prescription rates of antidepressants. I argue that depression is strategically suppressed by the military junta because they see it as a form of social unrest and protest.

My argument has seven parts: (1) Counting depression: The government claims that the rate of depression is 0.00006%, which is implausibly low. Other data say the same. The available statistics appear to confirm healthcare professionals' perceptions of low rates of mood disorders. (2) Psychiatric infrastructures: The military regime keeps the country's healthcare infrastructures weak, but not weak enough to explain low rates of mood disorders. (3) Repression without depression: There are many processes that should contribute to high rates of mood disorders, including alcoholism, domestic abuse, economic hardship, natural disasters, religious strife, and decades of government brutality. Burmese healthcare professionals recognize these processes, but they still think that mood disorders, in the narrow sense of psychiatry, are rare. (4) The psychopharmaceuticals market: A lack of availability and affordability of medications cannot explain why mood disorders are not treated more often. (5) Myanmar depends on importing pharmaceuticals because there is no encouragement for domestic producers. (6) Within the dictatorship’s sustained “drug elimination” regime, any substance classed as "psychotropic" is seen with deep suspicion. I conclude by arguing that (7) the dictatorship actively downplays the prevalence of depression. Myanmar's statistics are so different from other countries because the military regime is muting expressions of dissent while amplifying non-medical remediations of distress.

The trauma of military rule continues to shape healthcare. The country’s decade of quasi-civilian rule from 2011 to 2021 neither diminished the military’s control of business, politics, and the media, nor its control over healthcare. The military's dominance since 1962 has created a near-permanent “state of exception” where violence and surveillance became normalized aspects of daily life. The junta's approach to governance has always emphasized projecting an image of stability and hard-line control precisely because it faces so much resistance on so many different fronts (Aung & Campbell, 2024). The military's key goal, to maintain its iron grip, influences all aspects of public administration, including how symptoms are diagnosed and treated. I argue that, in Myanmar, mood disorders are seen as signs of political instability and protest. The standard argument in medical anthropology is that psychiatry often tends to medicalize socioeconomic distress (Kleinman & Good 1985; Wu, 2021; Lovell & Oppenheimer, 2022). Myanmar presents a radically different situation: here, dictatorial politics strategically suppress medicalization.

My interview with Dr. Zaw, an experienced clinician, took place in a busy private clinic in downtown Yangon (Rangoon) in January 2020. The clinic is located opposite the Yangon General Hospital, the country's largest hospital, founded in 1899 under British colonial rule. Dr. Zaw’s clinic offers medical consultations with specialist doctors, as well as a range of diagnostics, including gastroenterological examinations. To see a doctor costs US$10, full colonoscopies cost US$200. Most clients come for digestive problems. Dr. Zaw said that psychopharmaceuticals are occasionally prescribed in her clinic. Tranquillizers were used "for patients with insomnia," but not routinely. The patients coming to the clinic suffered from physical problems, and these problems were definitely not somatized distress. Dr. Zaw said that depression existed, but that the overall number of patients was very small. Other health problems were far more prevalent. Diabetes, chest infections, or hypertension far outnumbered any mental health issue. People's awareness of depression was also low, "they don't think they have depression" and therefore, they did not seek treatment. Depression had murky, fuzzy symptoms, she said. Clear types of "madness," such as schizophrenia, were far more distinct and recognizable. While lay awareness was low, getting help when seeking it was not a problem. Finding a psychiatrist was easy, Dr. Zaw said, especially in Yangon. Overall, depression was simply not a problem in the country.

**Fig. 1:**
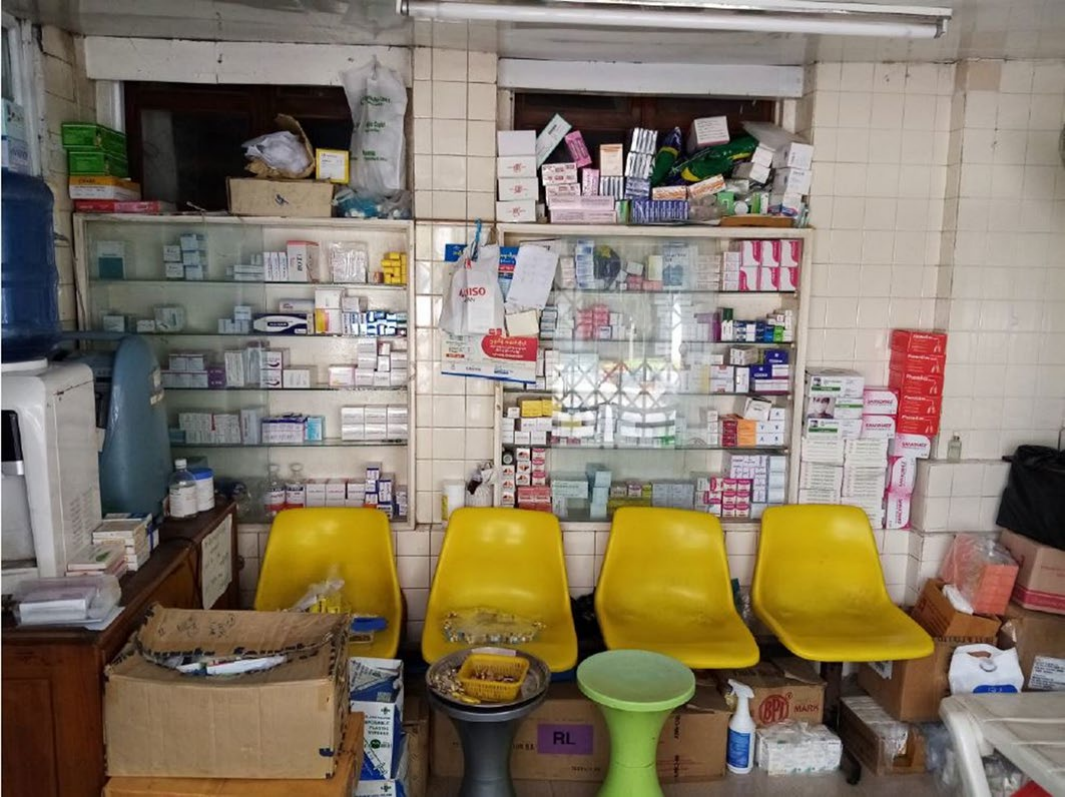
A GP practice in Yangon

Dr. Zaw's perception of low depression rates in Myanmar was typical for how healthcare professionals talk. Their perceptions were starkly different from India, where doctors say that depression is increasing rapidly. In previous research, I studied psychopharmaceutical uses in India (Ecks, 2014, 2022), tracing these medications from production to consumption. This included interviews with pharmaceutical producers, distributors, retailers, and various prescribers, as well as participant observation in clinics and pharmacies. I studied how affordable and accessible psychopharmaceuticals are, who the key producers and prescribers are, and for what problems the drugs are used. In further research, I followed these drugs from India to countries that import them. This is how I came to Myanmar (formerly Burma), an Indian Ocean country of 55 million people bordering India, Bangladesh, China, Laos, and Thailand. Most of the medications used in Myanmar are Indian generics. Yangon, the former capital, is the country's largest city and business center. All pharmaceutical companies are headquartered in Yangon, and all the major hospitals are in the city. Employing the "tracing pharmaceuticals" methodology I had used in India, I interviewed a variety of experts, predominantly in Yangon but also in a district town 200 km east of Yangon. These included pharmaceutical company executives (5), pharmacists (5), psychiatrists (5), general physicians (4), and a public health researcher. I use pseudonyms for all respondents to protect their privacy. All respondents spoke English, so I required no help with translations. I also carried out observations at the Yangon General Hospital, the Yangon Mental Health Hospital, at private hospitals, and at pharmacies (Fig. 1).

This study focused on interviews with English-speaking healthcare professionals and industry representatives in Yangon. While this approach provided important insights into institutional perspectives on mental health, it has important limitations. People living outside Yangon and ethnic minorities are underrepresented. The study's respondents represent an elite perspective, coming from educated, urban professional backgrounds. My data collection in Myanmar was impacted by the COVID-19 pandemic in 2020 and the military coup in 2021. Since early 2020, it has been effectively impossible to do fieldwork in Myanmar. What I am presenting here reflects a specific moment in time: nearly 60 years of military dominance partially punctured by democracy, shortly before the country’s return to full-fledged dictatorship in 2021.

My analysis is based on a multimediation model derived from living value theory (LVT), which extends an embodied theory of value (Ecks, 2022) through a deeper understanding of how value creation is structured. In LVT, health is the dynamic capacity of living beings for flourishing across multiple mediations and temporal scales.

Health is the dynamic capacity of living beings for flourishing across multiple mediations and temporal scales. Health is restored and enhanced in five major mediations: (1) *Sensing*: Embodiment is multisensorial, including—but far exceeding the main five senses, encompassing other ways of sensing such as interoception, chronoception, and nocioception. (2) *Being-with*: living beings, human and nonhuman, live in multispecies socialities. They live with each other and value each other in different ways. (3) *Forming* (multimaterialities): objects, tools, instruments, architectures, and built infrastructures are made to make live better. (4) *Dwelling*: Living beings inhabit multiple, overlapping worlds simultaneously—geologies, geographies, climates, and cosmic dimensions afford different ways of multiversal dwelling. (5) *Signing*: Multisymbolism allows for the encoding, expression, and communication of values. This comprises all forms of communication and sharing of information, including printed documents, visual and audio media, as well as digital platforms: internet, AI, and social media.

Where remediations afford uninterrupted *being-in-the-worlding*, there is no consciousness of health (Gadamer, 2018). Health is most fully present when it is most fully mediated, that is, when sensory, material, and social flows are integrated. Rather than a fixed state or a return to a stable "normal," health is an ongoing metabolic flow, in differential temporalities and changing contexts.

Biomedical therapies draw different kinds of mediations together, for example, antidepressants are symbolic-material mediations that alter multisensorial and social experiences. In LVT, pharmaceutical remedies appear as limited types of remediation, among a myriad of other remediations of health. The model backgrounds professional healthcare so that other, less obvious remediations can come to the fore. Multimediation is a continuous process of amplifying and muting, remediating and demediating. Remedies-as-mediations are amplified if experienced as health-enhancing, or muted when they do not improve health. Health processes can also demediate particular substances: this is what de-addiction therapies do. Since health is so deeply multimediated, there is ample potential for cross-mediations, which include substitutions and compensations. Psychopharmaceuticals, as material mediations, might be prescribed to compensate for disturbed multisocial mediations.

In the case of the low depression rates in Myanmar, the multimediation model looks for explanations both within and beyond the health sector. The reported rates could be low because the epidemiological data are unreliable. The rates could be low because mental health professionals are lacking. There could be problems with the availability, pricing, or importation of drugs. Beyond remedies as mediations, there could be other mediating processes, like political tensions, class relations, social ties, religious dynamics, gender, and marital relations. I argue that the low rate of reported depression emerges from the military’s complex multimediation of diagnostics, psychotropics, policies, infrastructures, and political propaganda.

## Counting Depression

My data consist of statistics, observations of material practices, and local expert interviews. Different data can be congruent whenever the statistics show a disease as prevalent, observations find that treatments are common, and interviews with experts confirm that both disorders and treatments are common. In the case of Myanmar, deep incongruences between different sets of data suggest that a form of "strategic ignorance" is at work (Gross & McGoey, 2015).

Globally, the rate of depression is 3.4%. Government statistics puts the rate of depression at 0.00006%. To be fair, all epidemiological data on mental health generally suffer from a "credibility gap" (Patel, 2014). Global burden of mental disease estimates works with small and outdated datasets, and much has been said about the unreliability of the diagnostic categories used to produce epidemiological estimates. Epidemiological studies, especially on countries in the Global South, consistently fail to state how weak the data are (Brhlikova et al., 2011). Nevertheless, the enormous difference between global and Myanmar prevalence statistics is clearly very odd.

The global rate of people living with any mental illness is 10.7%. The rate of depression is 3.4%. Other conditions are anxiety disorder (3.8%), schizophrenia (0.3%), alcohol use disorder (1.4%), and drug use disorders excluding alcohol (0.9%). Substance use and mental disorders combined affect 13% of people worldwide (Ritchie & Roser, 2020). Depression is the leading cause of long-term disability. The global ratio of schizophrenia to depression is 1:10; the ratio of alcoholism to depression is about 1:2.5. The global data are not nearly as robust as they should be (Patel, 2014; Rose, 2018; Shorter, 2013), but they can serve as a benchmark for Myanmar.

The epidemiological data on Myanmar mental health are exceptionally weak. In some WHO publications, Myanmar is said to have “similar” burdens of mental, neurological, and substance use disorders as other countries in the region (e.g., Nguyen et al., 2018; WHO, 2006). Global Burden of Disease (GBD) research puts anxiety and depression in the top ten worldwide (Institute for Health Metrics and Evaluation 2024). The leading causes of disability in Myanmar in 2017 were diabetes, headaches, low back pain, blindness, and chronic obstructive pulmonary disorder. In a review of the mental health literature on Myanmar, Nguyen et al. refer to GBD data and claim that a "large burden of mental health problems in the country is not surprising" (ibid.) because of violence and natural disasters. But they also mention that "most prevalence studies of MH problems have been conducted among expatriates from Myanmar" (2018, p. 2). Furthermore, none of the Myanmar studies included in GBD analyses is actually about mental conditions, they are just general health surveys. Extrapolating findings from traumatized refugee groups or from generic health surveys does not allow conclusions about mental illness burdens in the country. An online questionnaire posted on Facebook in the midst of the military coup and the COVID pandemic (Saw et al. 2023) showed astronomically high rates of mood disorders: 61% of respondents reported symptoms of depression and 58% had symptoms of anxiety. The authors believe that these rates are “probable” and not biased by the methodology.

**Fig. 2:**
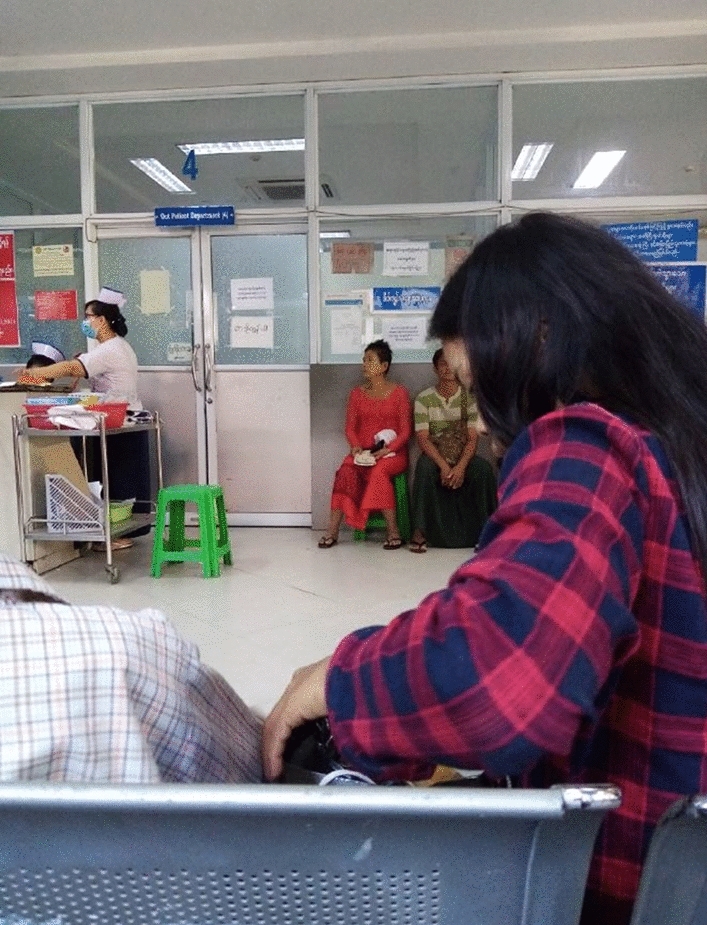
A psychiatric OPD in Yangon

A 2006 WHO report on Myanmar lists diagnoses of patients in the country's two mental hospitals: "mental and behavioural disorders due to psychoactive substance use including alcohol (34%), schizophrenia (28%) and mood disorders (27%)" (WHO, 2006, p. 6). Across all government mental health facilities, 9% of patients in outpatient departments (OPDs) were treated for mood disorders, 11% in "in-patient units," and 27% in mental hospitals. It is not clear what "in-patient units" are. The report states that "in outpatient facilities, neurotic disorders and schizophrenia are more prevalent; while within in-patient units schizophrenia, substance abuse including alcohol abuse are most common; and in mental hospitals, schizophrenia and affective disorders are most frequently treated" (WHO, 2006, p. 11). But the numbers contradict this claim: "schizophrenia" (12%) is ahead of "mood disorders" (9%) even in outpatient care. Rates of mood disorders are implausibly low (Fig. 2)

The government's statistics also state low rates of depression. The *Public Health Statistics Report 2014-2016*, the most comprehensive report available from the government, says the following (all grammar mistakes are in the original): "Mental health problem per 100,000 population in 2016 revealed that 9 person reported with psychosis, 6 person had depression, 7 person showed anxiety and mental retardation, 5 stayed with epilepsy, 120 depend on alcohol" (Ministry of Health and Sports, 2017a, 2017b, p. viii). In other words, the ministry says that the rate of depression in the population is only 0.00006%. Nothing is said about the methodology used. That psychosis outnumbers depression is odd. That mental illnesses are so rare is odd. That depression rates could be *56 times lower than in the rest of the world* is very odd.

A study on diagnoses given by psychiatrists in three community mental health clinics (Myint et al., 2020) records *ten times more psychosis than depression*. The study analyzes psychiatric case notes from three community clinics in periurban Yangon. The categories are depression, psychosis, epilepsy, child/adolescent mental and behavioral disorders, dementia, and substance use disorders. In 2017, psychiatrists diagnosed a total of 15,158 patients in these three clinics. Psychiatrists found 11,396 cases of "psychosis," which is more than 75% of all patients. This is an extremely high rate compared to global figures. The second most common problem is substance use, with 1377 cases. Depression scraped into third place with 1142 cases. Epilepsy came in at fourth place, with 877 cases. One of the three clinics is at Hlaing Thar Yar, which has a large population of forcibly resettled people. The psychiatrists diagnosed 4,684 cases of psychosis compared to only 474 cases of depression. Compared to other studies on symptom presentation in community settings, the rates of psychosis are astoundingly high. Elsewhere in the world, depression always outnumbers psychosis.

The epidemiological data were so puzzling that I specifically interviewed various health professional on them. In conversation, one of the authors of the Myint et al. (2020) paper confirmed that the study was a statistical analysis of psychiatric case files. The researcher's explanation of why psychosis is ten times higher than depression is that doctors use "psychosis" as an "umbrella term" that includes a range of mental health problems. "Depression" was only used for "clear cases" that have only symptoms of depression and nothing else. When I discussed this study with one of the country's most senior psychiatrists, he argued that the preponderance of psychosis in these three specific suburbs of Yangon could be explained by many local residents being previous inmates of the psychiatric hospital. While this study focused on community presentations of mental illnesses, "this is not a community presentation" elsewhere in the country. He also confirmed that there are no good large-scale studies on mental disorders in Myanmar. He said that the Ministry was planning a larger survey on mental health within the national health survey. The focus of this survey would be suicide and alcohol use. However, both these caveats, about psychosis as umbrella term or samples not being representative, are not enough to make sense of the exceptionally low rates of depression.

## Psychiatric Infrastructures

Just as there are no quality studies on prevalence, there are no good studies on mental health services in Myanmar. According to the *mhGAP Action Programme*, the number of Myanmar's health providers per 100,000 population (at 1.34) is slightly smaller than India's (at 1.87), but the number of mental health professionals is higher in Myanmar (1.11 compared to 0.31). In 2016, about 200 psychiatrists were active in Myanmar (Brennan 2016). The country's psychiatrists are organized in the Mental Health Society, which is part of the Myanmar Medical Association.

**Fig. 3:**
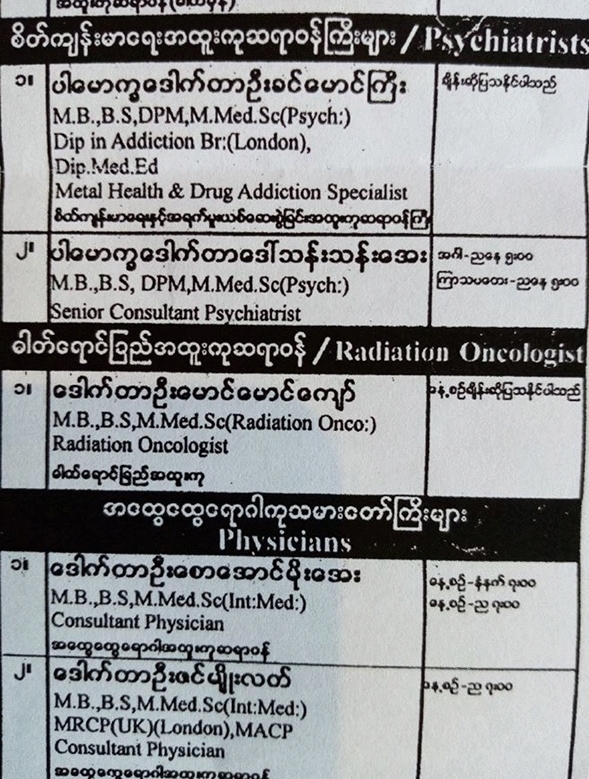
Leaflet advertising physicians’ consultation hours

Psychiatry is practiced both in the public and private sectors. Almost all the psychiatrists with government positions also work privately. Yangon has dozens of private mental health clinics. Yangon also has dozens of private medical centers and hospitals that have two or three psychiatrists on their rosters. The private hospitals list the doctors practicing there on billboards and on paper leaflets. I collected dozens such leaflets. In each center, at least two psychiatrists were available among the pool of specialists. Almost all the psychiatrists called themselves "drug addiction" experts. (Fig. 3)

Beyond psychiatry, a handful of private counseling services are available in Yangon. All of them have staff trained in the UK or the US because clinical psychology cannot be studied in Myanmar (Downing, 2016). Most clients are business expats and international NGO employees. Psychiatrist Dr. Htin told me that one of Yangon's psychological services is run by a member of a business dynasty close to the military. The family's businesses include arms trading and pharmaceuticals. A recent report by the UN Human Rights Council names the family as one of the top corporate donors to the Myanmar army, effectively bankrolling the warfare against ethnic minorities. The "consistent patterns of serious human rights violations and abuses in Kachin, Shan and Rakhine States" are impossible without funding from "crony companies" (UNHRC, 2019, p. 3). The owner of the psychological service divides her time between running the group's “social responsibility” arm and giving TED talks on how to deal with posttraumatic stress.

Public sector psychiatry reaches back to the British colonial era, which lasted from 1824 to 1948. Key institutions, such as the Yangon General Hospital and the Yangon Mental Health Hospital, were founded in colonial times: "Burma was a colony that, politically, economically, and militarily, was intimately linked to the British Empire, especially British India" (Taylor, 2015, p. 4). Burma was ruled as a province of British India until 1937, then as a separate province of the Empire until Independence in 1948. In 1886, the British opened the Rangoon mental hospital as a "prison for the insane" (Zaw, 1997). After WWI, a new mental hospital was built at Tadagalay, a village outside Yangon. "Tadagalay" became Burma’s idiomatic equivalent to "Bedlam," a scary place where mad people are sent to. Psychiatrists at the hospital also have appointments at the Department of Mental Health, University of Medicine 1. Psychiatrists and mental health nurses first trained in India and later also in Britain. The "father of Burmese psychiatry," Dr. U Ne Win, was the first Burmese psychiatrist to study in Britain before returning to the country in 1951.

**Fig. 4:**
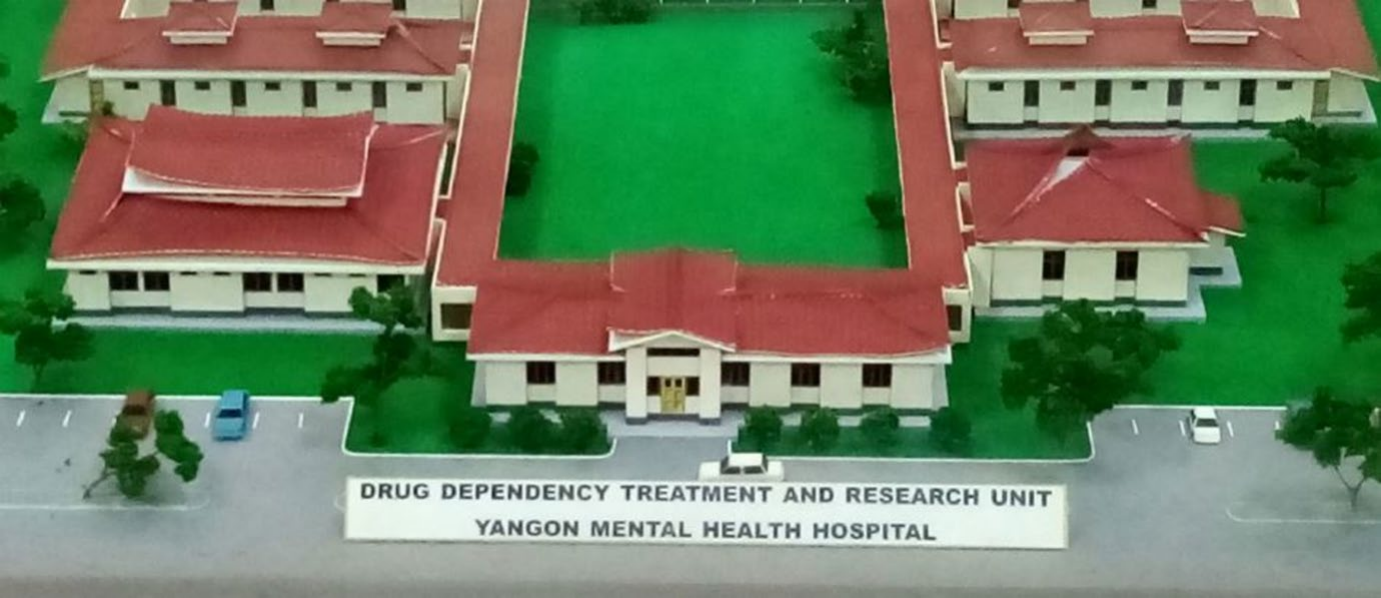
Model of Yangon Mental Health Hospital at the Drug Elimination Museum

In 2000, Tadagalay closed down and Ywar Thar Gyi Mental Health Hospital opened at a site 30km outside of Yangon. The hospital has 1,200 beds and an outpatient department. The hospital admits around 2,000 patients per year and treats 19,000 OPD patients (Myint et al., 2015, p. 293). Its sprawling campus has separate buildings for treatment groups (e.g., alcohol and drug addicts) and treatment types (e.g., electroconvulsive therapy). A senior psychiatrist told me that the Ministry chose the isolated location to let patients "enjoy the peace of the countryside." Many nurses and doctors have to reside on the isolated hospital campus because commuting from Yangon is arduous. One of the hospital's psychiatrists said that he spends two hours every day in the car to reach the hospital. A second mental hospital started out in Mandalay in 1966. In Yangon, the second largest psychiatric outpatient clinic is at the General Hospital, where patients are seen twice a week for a few hours. Psychiatric community clinics have run in various places, especially in periurban areas of Yangon (Myint et al., 2015) (Fig. 4)

Myanmar's health system is weak and receives very little government funding (Ergo et al., 2019; Latt et al., 2016). In the 2000s, Myanmar was ranked as *the worst* health system in the world (Tandon et al., 2000). Mental health is a low priority (Nguyen et al., 2018; Risso-Gill et al., 2014). Most of my respondents mentioned this as a reason why people are not seeking treatment. The only mental problems that the government seems interested in are substance use, domestic violence, and psychosis.

During the transitional period 2011–2021, the government increased its health spending but is still far behind other countries in Southeast Asia (Myanmar Healthcare Consulting, 2020). Many of my Yangon respondents pointed out that infrastructures had improved since 2011. For example, pharmacist Mr Pe said that the Aung San Suu Kyi government increased the health budget more than any previous government. An Indian pharmaceuticals manager, Mr. Iyer, said that the democratically elected government had "good intentions" and was "doing quite a good job, especially when compared to India."

In his brief history of Myanmar psychiatry, Zaw (1997) describes how, in the 1990s, the then-existing outpatient clinics in Yangon were overrun by patients: "The numbers seen at these out-patient clinics were huge! These two clinics were appointment clinics with morning and afternoon sessions, but whoever showed up without an appointment was also seen the session ending with the last patient who turned up" (1997, p. 507). After repeat visits to the psychiatric OPD at the Yangon General Hospital, I cannot confirm that these long queues of patients still exist (Fig. 1). Each time I visited, there were no more than 12–15 patients for each 2–3 h session, twice a week. I also went repeatedly to a large Yangon private hospital that GPs said they are referring their patients to for psychiatric treatment. The hospital had three psychiatrists on its roster, and they were scheduled to attend to patients for a total of 13 hours every week. However, each time I came, the doctors had either left early because not enough patients had turned up, or they did not come at all because no one had scheduled an appointment. Myanmar's rural areas are probably places "where there is no psychiatrist" (Patel, 2003). But in Yangon, there is no significant mental health treatment gap.

## Repression Without Depression?

In Yangon, depression is not a topic. All the healthcare professionals I interviewed felt that depression is of no concern. The psychiatrists said that depression is relatively common, but even among them, other issues are considered far more important, especially domestic violence and alcoholism. Dr. Maung, a GP who has practiced in Yangon for more than thirty years, said that “colds, fevers, chest infections, heart disease” made up "99 percent" of all his cases. All the pharmacists and pharmaceutical industry people said that the antidepressant segment is very small and stagnant. With the pharmacists I interviewed, I often had to ask about antidepressants by generic name before they even knew what I was talking about. For example, I talked to Mr. Kaung, the head pharmacist at a large medicine shop located across from the main entrance of the Yangon General Hospital. When asked about depression, he said “not a lot” had it. When asked about "antidepressants," he first said that "we have none at all." When I asked again, he confirmed that they did not have any. Then I probed with the names of molecules, such as "fluoxetine" or "amitriptyline." Only then did he list clozapine (an antipsychotic), alprazolam and diazepam (benzodiazepine tranquillizers), and fluoxetine and sertraline (SSRI antidepressants). These drugs made up just "one percent" of his shop's inventory. His customers preferred "the *necessary* drugs," such as antidiabetics and antihypertensives. Psychotropics were not "necessary.”

My Yangon respondents cited many reasons why people *should* be more depressed. They talked about the combined impact of pollution and traffic congestion as stressors. Yangon traffic is terrible and the air is badly polluted. Many mentioned that it used to be easier to live in Yangon before car traffic grew exponentially in the 2010s. A psychiatrist said that Yangon has such awful traffic because motorcycles were banned in the city in the 2000s, allegedly because military rulers in cars hated being overtaken by civilians on motorcycles. Violent street protests and road blocks made no difference, traffic was always bad.

Alcohol use was often cited as the leading cause of domestic violence and household poverty. Alcoholism was widely seen as the country’s top health disorder. The nexus between alcoholism and domestic violence is the most established trope. Dr. Myint, a psychiatrist at Yangon General Hospital, said that alcohol was "the number one problem in the country," and much psychiatric work was about alcohol detoxification. If anyone got depressed it was *because* they drank too much, not that people drank too much because they were depressed.

A survey of mental disorders treated in primary healthcare, January to September 2013 (cited in Myint et al., 2015, p. 292), counted 14,860 cases of alcohol dependence, 2024 psychosis cases, 1505 cases of mental retardation, 1472 cases of anxiety disorder, 1175 cases of depression, and 961 epilepsy cases. The Myanmar government's statistics on "mental health" argue the same: per 100,000 people, 120 were alcoholics, only 6 were depressed (Ministry of Health and Sports, 2017a, 2017b).

The alcoholism/domestic violence nexus has been extensively studied. Bjertness et al. (2020) explored the prevalence of domestic violence among 18–49 year old people in Yangon region over the past 12 month period. They found rates of domestic violence of 51% among women and 38% among men. Their definition of "violence" was adopted from the WHO's Demography and Health Survey, which includes emotional, physical, and sexual violence. Emotional violence was most common, followed by physical violence and, in third place, sexual violence. Lifetime prevalence of any form of domestic violence was 58% among married men and 41% among married women. Another study found that over 20% of people in Yangon region report mental distress (Aye et al., 2020). This includes anxiety and depression, but is much broader. A mental distress rate of more than 20% sounds high but is actually quite low compared to most other countries (including Canada, Cambodia, and India). In Kashmir Valley, which has also seen decades of political violence, people report 45% mental distress (Housen et al., 2017).

Since coming to power in the early 1960s, the military has violently suppressed dissent. Monique Skidmore, an anthropologist who explored mental health in Myanmar in the 1990s, describes fear as overwhelming and all-pervasive. Keeping one's head down became a survival strategy: "Burmese people have become experts—under monarchical and feudal rule, British colonization, and military dictatorship—in convincing themselves that so long as they act and speak in a few limited ways, they are safe from the daily onslaught of fear-making generated by those wielding political power" (Skidmore, 2004).

My respondents confirmed that people had many reasons to be fearful. Mr. Iyengar, a country director for a large Indian company, highlighted how people had to surrender their passports and how protesters were murdered in the 1980s. He told me that, in 1988, General Ne Win's government canceled 25, 35, and 75 kyat banknotes because they had “unlucky” numbers on them, and replaced them with 45 and 90 kyat notes because he believed 9 to be the luckiest number. This destroyed people's savings overnight and triggered widespread protests.

Myanmar has been marred by continuous internal conflict since the 1950s. The systematic expulsion and murder of the Rohingyas is only one among many interethnic (and interreligious) conflicts (Ibrahim, 2018; Lehr, 2019). Myanmar also suffered many natural disasters. The country is severely affected by climate change, flooding, droughts, storms, and earthquakes. In 2008, cyclone Nargis killed about 140,000 people. Nargis was the deadliest cyclone ever to hit the northern Indian Ocean (Paratharayil, 2010).

Decades of military dictatorship would appear to be unanimously bad for mental health, but many of my respondents described them as *good* for mental health. They said that the dictatorship can be an education in “respect for authority.” Submitting to authority freed people from having to think through everything themselves, and that was very good for mental peace. The country's isolationism under the military protected people's minds from getting confused.

Many highlighted Buddhism as health-enhancing. Theravada Buddhism is practiced by the vast majority of the population. The Buddhist concept of *dukkha* (suffering) frames life as relentless pain. In Buddhism, mental illnesses are often interpreted through karma, where current suffering results from past misdeeds. Buddhism influences both coping mechanisms and help-seeking. Many first turned to Buddhist meditation, making merit, chanting prayers, or consulting monks before seeking medical help. My respondents said that Buddhist concepts shaped how symptoms were understood. Depression was *seik nyit tae* (“contaminated mind”) or *seik shote tae* (“chaotic mind”). Some psychiatrists say they acknowledge Buddhist beliefs in treatments and recommend temple visits and meditation. The dictatorship portrays itself as heirs to precolonial kingship, as the *dhammaraja*, “righteous ruler,” at the center of a galactic polity (Schober, 2017; Tambiah, 2013).

Many saw the nexus of cultural traditions, religious practices, and dictatorial suppression as making people’s mental health better. Even Indian expats echoed this. In Myanmar, people still did what they were told to do, instead of contesting everything all the time. People's submission to authority was expressed in their unquestioning adherence to medications prescribed by doctors. The revival of democracy had not changed the high regard for authority figures. Established ways of life were still strong. The children had a good social life and adults spent a lot of time relaxing. My Indian respondents also said that the Burmese rarely had bank accounts (only 25% of the population had any kind of formal banking) and they also rarely had credit cards (one percent of the country's population had one). Not having banking or credit cards shielded against debt, and not having to worry about debt shielded against depression.

Bad economic policies and corruption made Burma one of the poorest countries in Asia. In 1987, the UN classified Burma as one of "the world's least developed countries." In the 2010s, the economy improved (World Population Review, 2020). After decades of social, political, and economic crisis, the country's brief return to quasi-civilian rule brought rapid change. The influx of investment from abroad trigged economic "gold rush" (Mr. Iyer). This has created new opportunities, but also new stresses. Mr. Rama, a country director for a large Indian pharmaceutical company, linked the small size of the Burmese psychopharmaceuticals market to cultural traditions, household finances, military dictatorship, and Buddhist meditation: "Psychopharmaceuticals is a very small market in Myanmar. Their mental health is much better. They don't have credit cards. They don't have debts. They don't have pressure. They are very humble, they do meditation, they are carefree. Tomorrow they may not have a job, they are not bothered. There is a huge difference in mindset to India. They have been conditioned under dictatorship for decades. They have not been exposed to the world. The culture is still intact. They respect their elders." Such characterizations cannot explain decades of protest against the regime. But these claims are not entirely naïve either. The military dictatorship is engaged in a complex multimediation of control, which includes the strategic co-optation of domains outside of institutional healthcare in the name of enhancing the health of the body politic. The amplification of “respect,” Buddhist practices, and health problems deemed to be apolitical, coupled with the muting of overt expressions of disagreement or of private banking so that people do not have to worry about financial debt makes perfect—if perverse—sense within the junta’s value cosmology.

## The Psychopharmaceuticals Market

**Fig. 5:**
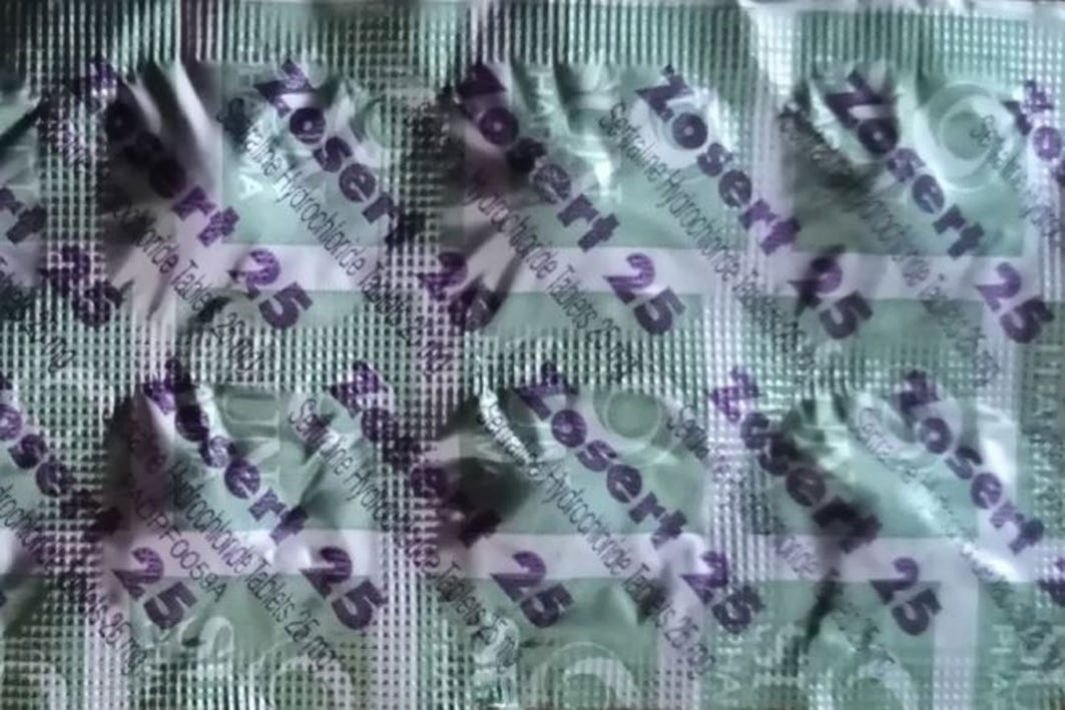
Generic antidepressants from India

In Myanmar, a wide range of psychopharmaceuticals are available at low prices. For example, a daily dose of Zosert, a generic brand of sertraline (Zoloft) produced by the Indian company Sun Pharma, costs 90 kyat (US$0.06). By comparison, OTC cough lozenges (e.g., Degirol) are 125 kyat apiece. A handful of high-end pharmacies also have some original brand medications. Sertraline is available as Pfizer's Zoloft, at a high price. Mrs. Myoe's pharmacy in downtown Yangon stocked Zoloft at the price of 1,500 kyat (US$10.33) per tablet. So, branded Zoloft is 176 times more expensive than the Indian generic. The market for such expensive brands is small, but it exists (Fig. 5).

In India, the SSRI antidepressant fluoxetine (Prozac) continues to be the most widely used antidepressant, even decades after it was first released. In Myanmar, by contrast, fluoxetine sales declined, and the leading molecules are now two other SSRIs, sertraline and escitalopram (Lexapro). All my respondents mentioned these two drugs as the bestsellers, most putting escitalopram first. According to a senior psychiatrist, fluoxetine used to be the most common antidepressant in the early 2000s, with Sun's Prodep as market leader. Then other SSRIs arrived in Myanmar (first citalopram, followed by sertraline and escitalopram). Then, Sun Pharma had problems with its import license, and the Prodep brand disappeared for several years. During that time, escitalopram substituted for fluoxetine: it had a wider range of uses, it could be used with most patients, and its availability in pharmacies was good. Fluoxetine had a narrower range of uses and was less available. Many of my respondents said that fluoxetine is "old." A younger psychiatrist, Dr. Myint, said that he learned in medical school that fluoxetine is "only for children."

An older generation of antidepressants, the tricyclics, is rarely used in Yangon. Tricyclics continue to be popular elsewhere in Asia. But in Yangon, tricyclics were generally seen as outdated. They had far too many side effects. Doctors also had to be mindful that patients drink alcohol and tricyclics are dangerous when taken with alcohol. In turn, the SSRIs worked great, even for alcoholics and people with damaged livers. A GP in a district town 200 km from Yangon said he uses amitriptyline because it also takes care of pain. He said that he did not like using it for too many patients because of the side effects, and that it should better be prescribed by "mental experts."

Antipsychotic drugs are widely used in Myanmar because schizophrenia and psychosis are widely diagnosed. These drugs are also used to treat psychoses induced by amphetamines and other illicit drugs. Antipsychotics can be combined with SSRIs. The main molecule is olanzapine (Zyprexa). Eli Lilly & Co.'s US patent for Zyprexa expired in 2011, ending annual profits of US$3 billion. Indian companies have been making olanzapine before India’s new patent regime commenced in 2005, and generic versions have been in Myanmar for decades. According to Yangon pharmacists, the market leader for olazapine is Olavex, produced by Vexxa, an Indian company. Psychiatrists like to prescribe antipsychotics for mood disorders, despite serious side effects. As psychiatrist Dr. Swe told me, the most problematic side effect of olanzapine is weight gain. For this reason alone, many patients did not want to take the drug. In Dr. Swe's experience, other antipsychotics, such as quetiapine, were better because they did not cause the same weight gains.

Another group of medications used to treat mood disorders are the tranquillizers. The key drugs are the benzodiazepines alprazolam (Xanax) and diazepam (Valium). They also come in affordable generics, but they are more difficult to access than SSRIs. They can only be obtained by prescription, and pharmacies need to apply for a special license to be allowed to sell these drugs. Strict regulations are also in place for opioid painkillers, which are nonetheless widely used. I found them in all the pharmacies I visited. The most popular is tramadol as Dolotram, made by Sun Pharma.

Mrs. Thant, a pharmacist at the Yangon General Hospital dispensary, told me that the government give medicines without charge on prescription from a hospital doctor. In some cases, patients' relatives had to buy medicines from outside shops if the prescribed drugs were out of stock. She went with me through what is available from the dispensary. In stock were escitalopram, olanzapine, alprazolam and diazepam. Escitalopram was available as Nexito by Sun Pharma and as Citalex by Opsonin, a Bangladeshi company. One brand of olanzapine, alprazolam, and diazepam was available, all of them made by Sun Pharma. Mrs. Thant said that the majority of prescriptions come from the hospital's psychiatric OPD and rarely from other departments.

**Fig. 6:**
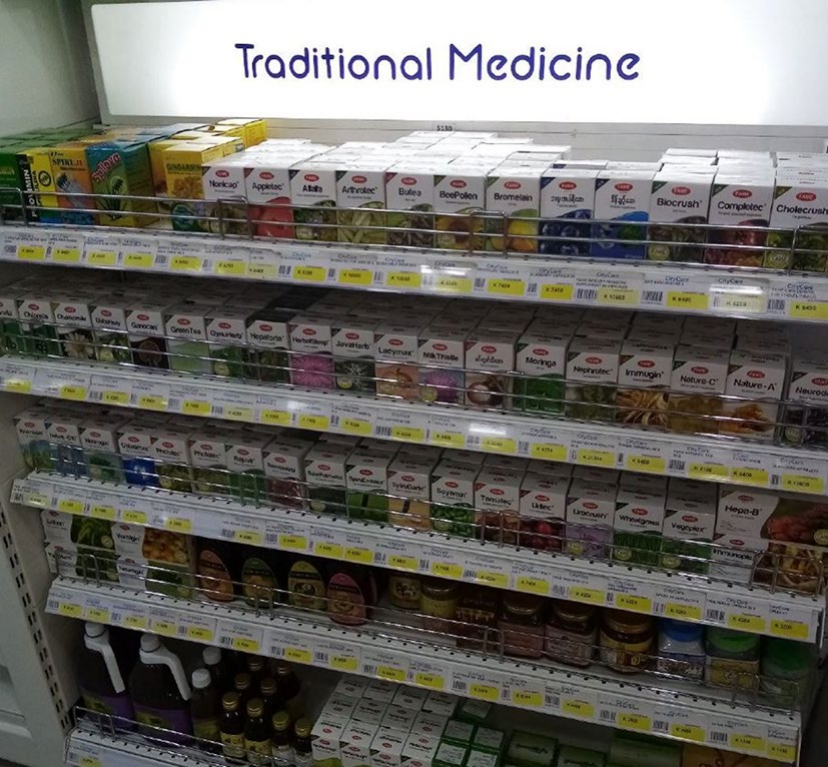
Traditional medicines on sale in Yangon drug store

It is also important to mention Myanmar's booming market in vitamins, minerals, herbal, and animal remedies. Advertisements for these OTC products are everywhere in Yangon. Many pharmacies have a "traditional medicines" section, which include substances promising to boost mental and physical performance. When I interviewed the pharmacist Mr. Kaung, he kept referring to these products as "placebos." In Yangon, "placebo" is the phamacists' term for "supplements." Common brands include Neurobion, a vitamin-B complex; Neurogin, which "stabilizes brain membranes;" and Neurodopa, which "replenishes the brain's dopamine." Edible birds nests are sold in solid and liquid forms. They are said to rejuvenate and to energize. Another bestselling product is "essence of chicken," which "sharpens the mind" and "fights stress." An "exam pack" consisting of 6kg of chicken extract is advertised to students. The market leader in essence of chicken, Singapore-based Brand's, runs "brain study camps." A CME (Continuous Medical Education) that I witnessed at the Mental Hospital was sponsored by United Pharmaceuticals, a Yangon-based supplier. After serving lunch to 50 psychiatric staff and medical students, the MRs gave a 30-minute presentation on three products: Enervon-C (C and B vitamin combination to "improve nerve function"), LiverCare (an extract of milk thistle to “tone the liver”), and Cholinerv (citicoline to "increase dopamin receptor density"). None of these is a "psychopharmaceutical" in the narrow sense, but psychiatrists routinely prescribe them for superior mental performance (Fig. 6).

## Import Dependence

While psychopharmaceuticals are about as affordable in Myanmar as in India, they are harder to find in Myanmar. There are fewer prescribers and pharmacies in the country compared to India. Many pointed out that Myanmar's population of 55 million is tiny compared to India's 1.3 billion. The Indian state of West Bengal alone has double the population of Myanmar. Furthermore, decades of economic stagnation had deepened economic inequalities. Indian pharmaceutical managers said that Myanmar "virtually has no middle class" (Mr. Iyer). A large middle class made the best customer base for pharmaceutical companies.

Because of a smaller base of prescribers and patients compared to India, the range of drugs that pharmacies stock is narrower. A Yangon pharmacist, Mr. Kaung, told me that he would not have more than two or three different brands of any particular molecule available in his shop. Another pharmacist, Mrs. Myoe, said that she keeps one or two, but at most three different generic brands of the same medication, any more is unnecessary. It would not make sense, she said, to keep too many of "the same" medications in stock. If a doctor writes a prescription for a particular brand that she does not keep, she would substitute it with a brand she has available. Yangon pharmacists said that many doctors write the active ingredient rather than a brand name. In India, by contrast, almost all doctors prescribe by brand, which requires pharmacists to keep a range of brands of "the same" molecule (Ecks, 2022). The pharma managers complained much about this difference between India and Myanmar because it made marketing difficult: "you cannot expect brand loyalty from the doctors here," said Mr. Pillai. Pharmacists said that some doctors also write on the prescription "must buy European" and name an expensive brand. He also said that Indian brands were only "medium effective." Patients who afford it tried to initiate treatment with a European brand before moving down to an Indian brand.

For all medications, Myanmar is entirely dependent on imports. The country has only a handful of pharmaceutical manufacturers, and most of them make herbal remedies and cosmetics. A new drug manufacturing plant opened by Alidac Healthcare in the Thilawa Special Economic Zone in 2018 was such a big step for the sector that several respondents mentioned it. By contrast, India is one of the world's leading producers of generic medications, which are both for export and for the domestic market. The Indian market is dominated by Indian manufacturers. India is Myanmar's leading importer (Myanmar Healthcare Consulting, 2020). Large Indian companies, such as Sun, Zydus Cadila, and Ranbaxy, supply the bulk of all products sold in the country. There was a certain anxiety in the pharmaceutical business about a high degree of bureaucratic red tape in license applications and in import regulations: "really, too much hassle." Regulations and policies could suddenly change: "Here in Myanmar, the policy can change overnight. Somebody says, 'it has to change', and it changes."

Myanmar also imports from countries other than India. My Indian respondents highlighted the growing presence of manufacturers from other Asian countries, especially from Pakistan, Bangladesh, the Philippines, and Korea. Some Asian manufacturers had a small advantage over the Indian companies because they joined the WTO's Agreement on Trade-Related Intellectual Property Rights (TRIPS) later than India. India became a full TRIPS member in 2005. Pakistan only acceded in 2010, Bangladesh in 2011. After TRIPS, medications that have patent protection cannot be made generically, unless a manufacturer pays a license fee to the patent holder. Companies from Pakistan and Bangladesh have a five to six years edge on their product ranges because they make drugs that were already out of reach of Indian companies since 2005. However, this specific advantage does not come to play in the psychopharmaceuticals market. Due to the industry's deep innovation crisis—all existing psychopharmaceuticals are decades old—the drugs prescribed today have been around long before TRIPS (Ecks, 2022). TRIPS has had no impact on the circulation of psychopharmaceuticals in Asia.

A proliferation of companies makes for "stiff competition" (Mr. Iyer). The pharma managers highlighted how low prices of drugs make business difficult for them. In Myanmar, the motto is "volume, not value" (Mr. Rama). Expensive medications do not sell well and competition in the low-price segment is strong. In the "gold rush" decade 2011–2021, many multinational corporations returned to Myanmar. But their expectations were disappointed and many scaled back their operations after just a few years. GSK, for example, closed its Yangon office in the mid-2010s. The company now manages its Myanmar operations from an office in Bangladesh.

More than the Indian companies, the US and European multinationals had to manage the risks of "reference pricing" (Mr. Pillai), that is, the risks of selling products at low prices. If buyers in high-value markets knew for how little money they could purchase "the same" medications in Myanmar, they could use this to negotiate lower prices with manufacturers. This is a risk of "information spillover—the knowledge about lower prices in developing countries generating demand for lower prices in developed ones" (Chaudhuri, 2005, p. 323). Multinationals have to strike a balance between the risks and rewards of being active in markets such as Myanmar's, and many decided that the risks outweigh the rewards.

**Fig. 7:**
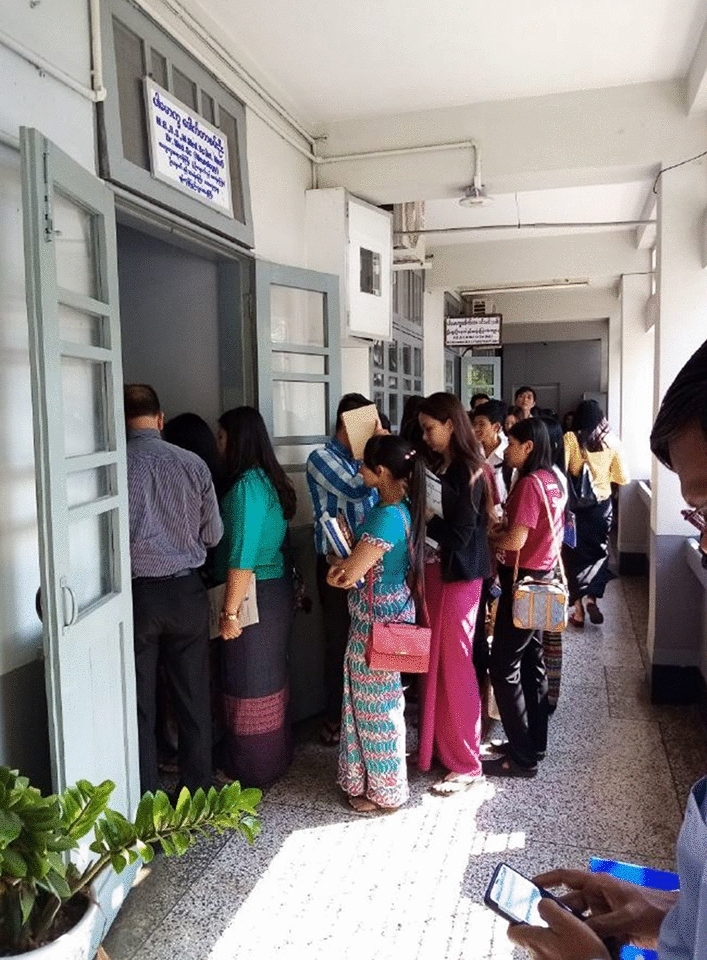
Yangon medical reps queuing to see senior doctor

The country manager of one of a top Indian companies said he focuses on "scientific marketing." Instead of competing on price, it paid to work closely with doctors and to convince them that not all brands were produced to the same standards of quality. But the largest share of the marketing efforts consists in influencing doctors through expensive gifts, meals, travel abroad, or direct cash payments. Marketing to doctors is just as aggressive in Myanmar as it is in India (Ecks, 2014). High-value prescribers are targeted by medical representatives (MRs). An MR gets only two minutes face time with a doctor to make his pitch. Larger clinics bundle the visiting times for MRs into dedicated time slots each week. Dozens of MRs form queues in front of doctors' offices (Fig. 7).

Another reason for the success of Indian companies in Myanmar—as opposed to the multinationals—was that they were better at "making do" even in a small, regulated, and politically fickle market. Indian companies had been in Myanmar for decades, "sticking it out" during years of socialism, military dictatorship, economic sanctions, and violent insurgencies. Enduring hard times was their strength. The western multinationals did not have the same “grit,” that is why they came and left again (Mr. Iyengar). The long-time presence of the Indians allowed them to build lasting trust among doctors. Patience paid off.

Pharmaceutical businesses also needed more patience than in India for getting licenses to import and market their products. All the pharmaceutical managers said that the licensing process was a major hurdle. Any pharmaceutical product needed a license from the Myanmar Food and Drug Administration (FDA). The application dossier was difficult to compile. After submitting the application, it took more than one year for the license to be granted. In India, the same application would take only three months. Another hurdle was the FDA's restrictions on how many licenses it grants per molecule. If there are already several products with "the same" active ingredient in the Myanmar market, the FDA would refuse new licenses. Some Indian importers, including some of Indian's leading psychopharmaceuticals producers, did not even bother to apply for licenses for all their products because of these restrictions. To be successful meant to be selective with their choice of products. The Myanmar market was simply too small to make it worthwhile to promote any product. One of the companies that is a market leader in psychopharmaceuticals in India never even applied for any licenses in this segment in Myanmar.

## Demediating drugs

Another reason for the small market was that the Myanmar FDA is more restrictive with anything regarded as "psychotropic." Tranquilizers and opioid painkillers are strongly regulated, both in market licenses and in retail. In practice, one can buy "by prescription only" medications over the counter. Yangon pharmacists I interviewed said that this is not allowed, but I went to three pharmacies and asked for various SSRIs, and could purchase them without trouble. But tranquilizers and opioid painkillers could not be bought without a prescription.

The government's tense relation to all things "psychotropic" has a deep history. Since the 1950s, Burma has been the world's leading exporter of opium, and since the 2000s also the top exporter of heroin and methamphetamines. During the decade of parliamentary rule, Afghanistan overtook Myanmar in opium exports but under the renewed military dictatorship, the country regained the world’s top position in 2023 (Yong, 2023). Until the 1950s, Burma used to be the world's leading exporter of rice. Then, the "narcoeconomy" became the country's most lucrative business. The Golden Triangle, the area between Burma, Thailand and Laos, is where opium poppies are cultivated. After WWII, Mao's China shut down Chinese opium production and expelled the anti-Communist Nationalists, and many of these Kuomintang troops moved into the Golden Triangle. With support from the CIA, the Kuomintang operated like a private narcotics army that controlled the borderlands and the drug trade. It took the Burmese army a decade to defeat the Kuomintang, but the control of the illicit drugs production shifted to other groups, such as the United Wa State Army. In the 1990s, the Burmese military stopped waging major operations against opium growers and instead took over the trafficking (Chin, 2011, p. 195; Bernstein & Kean, 1996; Brown, 1999).

**Fig. 8:**
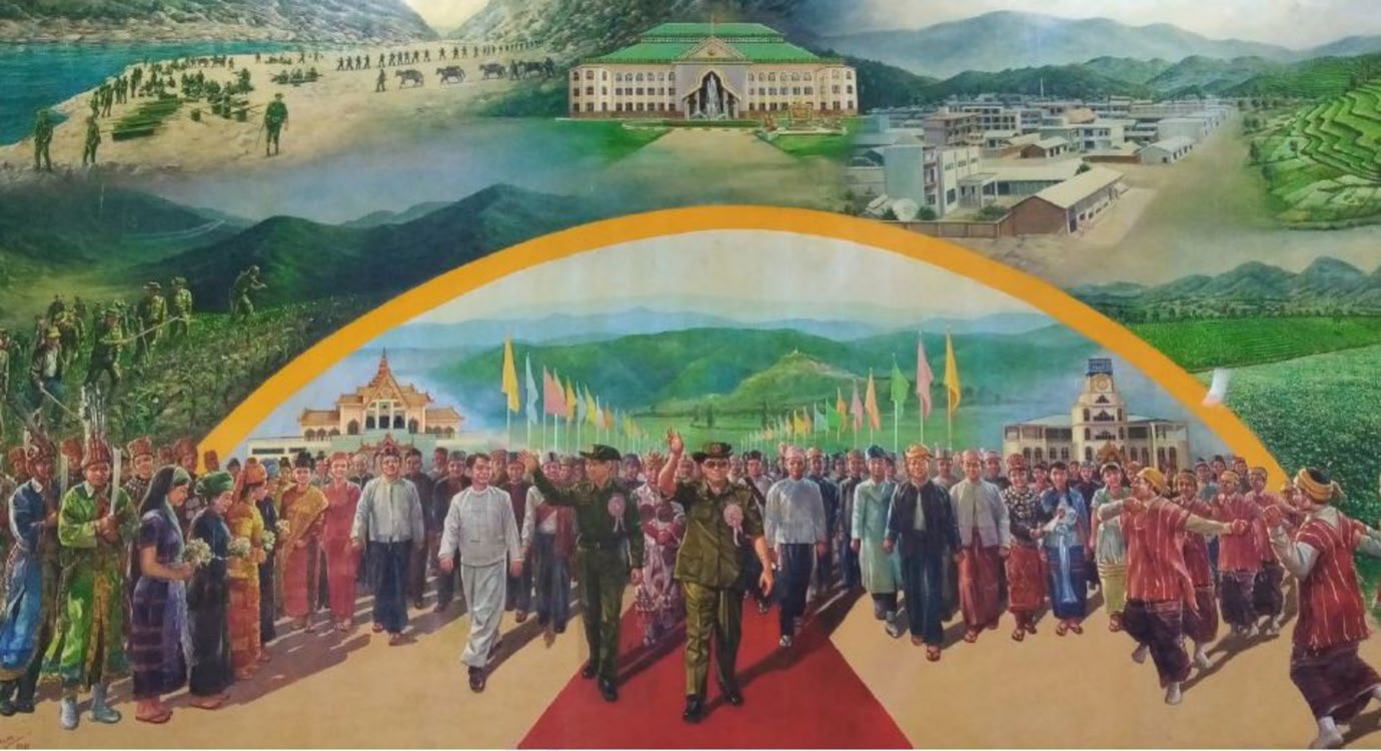
Panorama painting at the Drug Elimination Museum

Official policies are strictly against illicit drugs. In 1974, Myanmar's government introduced a new Narcotics and Dangerous Drugs Act in order to "combat drug abuse, an issue that threatened the entire Myanmar race." The Act includes that addicts must get registered and undergo compulsory detoxification treatment. In amendments to the Act (1983, 1988), the requirements of compulsory medical treatment of addicts were given even more severe penalties (Fig. 8).

The government leads a propaganda war against "psychotropics" in all their forms. For example, there are several "Drug Elimination Museums" across the country. The largest is in Yangon. It consists of a memorial, a park, and an exhibition building the size of an large shopping mall. On more than 14,300 square meters, the museum features planes used to bomb opium fields, life-sized dioramas on the degradation of Burmese society by drugs, and interactive displays such as a button that incinerates seized goods. The museum also has dioramas of how drug addiction is treated in the Yangon Mental Hospital, in outpatient clinics, and in "Rehabilitation Centres" that look like prison camps. It also has many displays that blur the boundaries between legal drugs and illegal drugs. Bottles of whiskey are placed with ecstasy tablets. Several cases entitled "psychotropic drugs" display packets of prescription medications such amitriptyline, alprazolam, diazepam, fluoxetine, and sertraline. One display item classifies "psychoactive drugs" as "opiates, hallucinogens, marijuana, stimulants, nicotine, psychotherapeutics, antidepressants."

The Drug Elimination Museums displays the dictatorship’s (de)valuation of drugs. The Museum presents a typology of drugs and traces a long genealogy of where drugs come from, how drug production became entrenched, how the dictatorship fought against production and distribution networks, and what a sustainable drug-free future looks like. The exhibition emphasizes that drug use “threatens the entire Myanmar race.” Drug users had been “looked down and shunned” since the Buddhist kingdoms. Much of the ground floor exhibition makes a single point: that “the habit of eating, drinking and smoking opium is not a Myanmar tradition, and was introduced into the country by foreigners from abroad,” as one display says. Drugs are a corrupting impurity, introduced by British colonizers. The massive expansion of drug production since the late 1940s is blamed on exiled Chinese and US Americans. The exhibition argues that drugs threaten to destroy the nation, and that the only way to fight off this disease is a multimediation that achieves both the elimination drugs and reintegration of producers and users. The strategy aims “to uplift and change the moral and perception of drug users and poppy growers. To secure smooth and easy transportation and communication between the nationals residing in highland areas and those in mainland. To uplift the economic and social life of the national races residing in the border areas.” For the dictatorship, drugs cannot be eliminated without compensating for the livelihoods provided by drug production. The demediation of narcotics must be combined with multiversal redress (building transport links to hilly areas), multimaterialities (industrial development), changed human/human relations (reintegrating opium farmers into the “national fold”), changed human/animal relations (“pig farms for alternative income generation in North Eastern Shan State”), and changed human/plant relations (“mushroom cultivation as a substitution crop for better opportunities”). Given the military’s involvement in the narcoeconomy, the museum can be dismissed as mere propaganda. And yet it puts on display how Myanmar’s authoritarian regime realizes that health is irreducible to transactive dualisms of prescriber/patient or army/opium grower.

## The Suppression of Depression

Is depression as rare in Myanmar as healthcare professionals and statistics say it is? The starting point of my exploration was the perception of Yangon doctors, pharmacists, and pharmaceutical executives. When Dr. Zaw told me she had "never seen anyone with severe depression," at first I thought she was an outlier, but it turned out that her views are typical among people in the Myanmar healthcare sector.

The available statistical evidence on mood disorders is extremely weak. Instead of saying that Myanmar *must* have extremely high rates of mood disorders because of so many different negative stressors, as all global health researchers outside the country do, it is better to admit that nobody knows. There is no way of knowing what the prevalence rates are. All the statistics are unreliable. How common depression is in Myanmar is anyone's guess.

Psychiatric infrastructure is relatively weak, certainly beyond the Yangon region. But it is not nearly weak enough to explain extremely low rates of depression diagnoses and treatments. In Yangon, trained doctors are in reach and medications are affordable. One of the country's most senior psychiatrists, Win Aung Myint, said that there are enough psychiatrists in the country, the problem is that people do not go to them: "We have treatment, but the public doesn't utilise it" (cited in Downing, 2016). If there is a treatment gap, it is not because there are too few doctors or drugs. In Yangon, no substantial gap between people seeking help and people receiving help is evident.

People in Myanmar could be expected to have many reasons for mental health problems: decades of political oppression, social inequality, interethnic warfare, religious conflict, domestic violence, excessive alcohol use, and natural disasters (Aye et al., 2020). There are plenty of reasons to be distressed, and my respondents were all well aware of many social, political, economic, and environmental factors that can contribute to higher rates of mood disorders. And yet, even with all these stressors present, neither perceptions of depression as being a leading health problem, nor the available statistics show that this is the case. Indeed, if people were living in crippling fear during the dictatorship years (Skidmore, 2004), then the decade of quasi-civilian rule should have reduced mental distress. However, there is no good evidence for this either. Finally, Myanmar hardly has any biomedical pharmaceutical manufacturers and relies on imports. But again, all the usual drugs are easily affordable. The government's war on drugs may treat legitimate drugs as if they were illicit, but psychopharmaceuticals are available.

A key explanation for low depression rates is the dictatorship’s direct control of healthcare, and indeed all sectors of society. That mood disorders are politically suspicious has been mentioned in passing by other researchers. Based on qualitative research on the role of Buddhism in Burmese ideas of depression, Schödwell, Steinhäuser, and Auckenthaler found that “the pervasive climate of fear and repression under the military government in the past decades meant that the open discussion of psychosocial conflicts was highly discouraged” (2019, p. 45), yet they do not link this to the low rates of depression in the statistics.

Psychiatrist Dr. Htin, who has worked with Rohingya refugees and comes from one of the ethnic minorities violently suppressed by the military, described severe censorship of mental health research. The army knew what violence it was inflicting on so many people for so many decades. He recounted many instances when research about trauma, depression, and anxiety were outright suppressed. Once Dr. Htin wanted to publish a study on trauma among refugees but was told he could not do that: "The Myanmar army, they make the people suffer. They know. And they are afraid to tell. Schizophrenia is not 'their fault', but trauma, anxiety and depression are. The government psychiatrists are very afraid to talk about trauma, anxiety, and depression. There is political, social, and economic suppression. Because most of the wealth of the country is still in the hands of the army. So, the Myanmar psychiatrists are *their army*."

According to Dr. Htin, mood disorders were suppressed because the military regime classed them as expressions of social and political distress. Among the psychiatric diagnoses, "psychosis" and "schizophrenia" were preferred because they were seen as purely biological, therefore politically innocuous. For brain-based disorders, the military could not be blamed. But for disorders caused by sociopolitical upheaval, the military could be blamed.

This interpretation of the link between mental illness and political dissent is the opposite of what has been said about the political connotations of schizophrenia in the US and in Europe. For example, Foucault's (2008) critique of psychiatry is that the discipline is a handmaiden of power that turns political nonconformity into medical pathology. In 1960s US psychiatry, psychosis was reframed as a disease of violent protest typical of African Americans (Metzl, 2010). In Myanmar, the opposite is true: instead of turning political dissent into medical disease, psychiatry separates brain-based disorder from political disorder. The military classes psychosis as a biological disorder that had nothing to do with politics. Hence, psychiatrists feel free to diagnose people as psychotic. Mood disorders, however, were caused by political distress, hence mood disorders were an expression of political protest.

The same coding of mental disorders by political etiology is typical for psychiatry in other socialist dictatorships, especially the Soviet Union (Bauer, 1952). Since the beginnings of Myanmar's “socialist way” in the 1960s, Burmese researchers were closely connected to scientists in socialist dictatorships (Holmes, 1967). In Soviet psychiatry, "the subjective factors of behaviour, particularly in the forms of neurosis, depression, or anxiety, were always vulnerable to being read as symptoms of social dysfunction. Far more ideologically preferable were clinical approaches that saw the psyche in materialist (psychoneurological) terms, and affective disorders as individual pathologies rather than signs of neurosis in an oppressive social world" (Matza, 2018: 38). This coding of mental disorders by political etiology goes a long way in explaining why psychosis is diagnosed far more often than depression in socialist dictatorships. Psychosis had a biological etiology, whereas depression had a political etiology. Psychosis came from bad biology, depression came from bad feelings about the state of society.

In 2014, Dr. Htin did a study on the prevalence of anxiety, depression, and trauma among medical staff in two Yangon clinics. Out of 400 participants, 20% reported symptoms of depression. When he presented his findings at a psychiatric forum, he met with fierce resistance by the other doctors: "a government professor got very scared and upset with me. 'That is not Myanmar result!' he shouted." He submitted funding applications to the government's Department for Medical Research (DMR) to do further research, but was rejected. "If you want to study depression, they will resist you." Dr. Htin also mentioned the government's mental health statistics, including the claim that the country's depression rate is "6 out of 100,000." He once discussed these statistics with WHO representatives. They were incredulous at the low rates and said that, clearly, "more research was needed." But the DMR did not support research into mood disorders.

To put the claim about the suppression of depression further into context, I checked other sources. DMR's Annual Reports list all research ethics applications received. The most recent report available (Ministry of Health and Sports, 2017b) has only three applications for mental health research: one on postpartum depression, one on the mental health of Kachin children, and one on lay awareness of epilepsy, psychosis, and depression. There was nothing on trauma and violence. The largest psychiatric conference ever held in Myanmar was the 18th International Congress of the Pacific Rim College of Psychiatrists 2018. It featured half a dozen poster presentations by junior researchers on depression. Two posters addressed posttraumatic stress due to violence. One study found high levels of emotional disturbance among children of military families (Khant, 2018). A study among adolescent depression found prevalence rates of 40% (Lin, 2018). Among working mothers in Hlaing Thar Yar, 76.8% of those classified as "labourers" were depressed (Thida, 2018). In the short decade of parliamentary rule, a few younger researchers started to study mood disorders. The military coup of 2021 returned to a systematic suppression of depression.
